# Decoyinine Induced Resistance in Rice against Small Brown Planthopper *Laodelphax striatellus*

**DOI:** 10.3390/insects13010104

**Published:** 2022-01-17

**Authors:** Amir Zaman Shah, Chao Ma, Yuanyuan Zhang, Qiuxin Zhang, Gang Xu, Guoqing Yang

**Affiliations:** 1 College of Horticulture and Plant Protection, Yangzhou University, Yangzhou 225009, China; dh18052@yzu.edu.cn (A.Z.S.); machaoyzu@163.com (C.M.); zhangyuanyuan182@126.com (Y.Z.); mx120190634@yzu.edu.cn (Q.Z.); 2 Jiangsu Co-Innovation Center for Modern Production Technology of Grain Crops, Yangzhou University, Yangzhou 225009, China; 3 Joint International Research Laboratory of Agriculture and Agri-Product Safety, The Ministry of Education of China, Yangzhou University, Yangzhou 225009, China

**Keywords:** decoyinine, small brown planthopper, induced resistance, fecundity, antioxidant enzymes, synthases enzymes

## Abstract

**Simple Summary:**

Small brown planthopper (SBPH, *Laodelphax striatellus)* is a serious rice sap-sucking insect pest in East Asia, especially in China. Furthermore, it is also a potential vector of rice viral diseases, such as rice stripe virus and rice black streaked dwarf virus, which cause significant yield losses. Here, a novel antimicrobial pesticide, decoyinine was applied as a seed treatment in rice in order to study life table parameters of SBPH and biochemical and physiological indices (having a role in induced systemic resistance in plants) of rice crop in response to SBPH feeding. Decoyinine significantly reduced fecundity in SBPH and also altered chemical and physiological indices of rice in response to SBPH. We conclude that the use of decoyinine in rice will contribute to integrated pest management (IPM) and may potentially provide a new idea for green technology.

**Abstract:**

Induced resistance against SBPH via microbial pesticides is considered as an eco-friendly and promising management approach. In this study, the induced resistance against SBPH in rice seedling by a new potential microbial pesticide, decoyinine (DCY), a secondary metabolite produced by *Streptomyces hygroscopicus,* was evaluated to investigate the effects of DCY on SBPH’s biological and population parameters along with defense-related physiological and biochemical indices in rice against SBPH feeding. We found that DCY has potential to improve rice resistance and significantly reduced the fecundity of SBPH. Laboratory results revealed that DCY treated rice significantly changed SBPH’s fecundity and population life table parameters. The concentrations of hydrogen peroxide (H_2_O_2_), soluble sugars and malondialdehyde (MDA) were significantly lower in DCY treated rice plants against SBPH infestation at 24, 48 and 96 hours post infestation (hpi), respectively. The concentrations of antioxidant enzymes, catalase (CAT) was significantly higher at 72 hpi, while super oxidase dismutase (SOD) and peroxidase (POD) concentrations were recorded higher at 96 hpi. The concentrations of synthases enzymes, phenyl alanine ammonia-lyase (PAL) was higher at 48 hpi, whereas polyphenol oxidase (PPO) concentration was maximum at 72 hpi against SBPH infestation. The results imply that DCY has unique properties to enhance rice resistance against SBPH by stimulating plant defensive responses. Microbial pesticides may be developed as an alternative to chemical pest control.

## 1. Introduction

*Oryza sativa* L. is a major staple cereal and also serves as a host for several pests that can greatly decrease rice yields [1,2]. The small brown planthopper (SBPH), *Laodelphax striatellus* (Hemiptera: Delphacidae), is a serious rice sap-sucking insect pest in eastern Asia, including China, Japan and Korea [3]. Leaves infested with SBPH turn yellow, wilt and even die, reducing yield and quality. Furthermore, SBPH is a potential vector of rice viral diseases, including rice stripe virus and rice black streaked dwarf virus, which also cause significant further yield losses [4,5,6]. Pesticides are commonly used to manage SBPH, which in turn results in the decline of natural enemies, environmental pollution, insecticide resistance and pest resurgence [7]. As a result, host-plant resistance has been identified as one of the most cost-effective and environmentally safe strategies for managing SBPH [8,9]. In recent years, the root microbiome emerged as a vital influence on various plant growth and resistance features [8,9]. Beneficial rhizospheric microbes can improve plant health by fixing atmospheric nitrogen, solubilizing plant nutrients which are unavailable in special types of soils such as rock phosphate and enhancing photosynthesis in plants [10,11]. Furthermore, these microbes enable the re-generation of damaged plant tissues upon herbivory due to enhanced photosynthetic activities, which in turn stimulates plant tolerance against insect pests [12,13,14]. Greater photosynthesis efficiency enables beneficial microbes to convert more light energy, which allows the generation of induced systemic resistance (ISR) against phloem feeding insects, which can compensate for the losses [15]. These microbes can also improve plant health in other ways, which include the production of secondary metabolites, enzymes, volatile organic compounds and growth hormones. All these directly or indirectly trigger ISR in plants against insect herbivores [16]. A rhizospheric bacteria, *Bacillus velezensis* YC7010, has antimicrobial, plant growth-promoting and systemic resistance-inducing activity against BPH via rice root drenching [16]. Colonization of rice roots by *Pseudomonas fluorescens* WCS374r induces ISR through enhanced accumulation of phenolic compounds [17]. *P. fluorescens* strains Pf1, TDK1 and PY15 display ISR against the leaffolder (*Cnaphalocrocis medinalis*) larvae by the activation of PPO in rice plants [18].

As a biocontrol agent, microbes and their byproducts offer an effective substitute to chemical control, which provide an efficient control with minor or zero ecological impact [19]. Plant root Rhizobacteria have a latent biological control effect [20]. This can form part of integrated pest management and has gained attention from researchers as a possible safe and environmentally friendly pest management option [21]. Recently, microbial insecticides have also attracted considerable attention [22] because they are more specific, have low relative cost and are more eco-friendly [23,24,25]. The biological control agents derived from actinobacteria, *Streptomyces* spp., are reported to be the most researched and well recognized genus of actinomycetes owing to their recognized importance in farming and health, functioning as biofertilizers and biocontrol agents by enhancing plant growth and antiherbivore resistance [26]. *Streptomyces* are Gram-positive, culturable, rhizospheric bacteria which produce secondary metabolites and phytohormones [27,28,29,30,31,32]. *Streptomyces* are well recognized and extensively reported for secondary metabolite synthesis, with a distinct structure and mode of action that provides a key option for biocontrol [33]. Biological activity of microbial secondary metabolites of actinobacteria, such as *Streptomyces* and *Streptoverticillum* against *Spodoptera littoralis,* caused larval and pupal mortality [34,35]. Secondary metabolites from *S. hydrogenans* DH16 showed deleterious effects on growth and development of *S. litura* larvae. A novel polyketide metabolite isolated from *Streptomyces sp.* AP-123 showed larvicidal and growth inhibitory activities against *Helicoverpa armigera* and *S. litura* [36]. Similarly, insecticidal activity of crude ethanolic extracts from *Streptomyces sp.* have been known to cause mortality of *Sitophilus oryzae* larvae [37].

**Figure 1:**
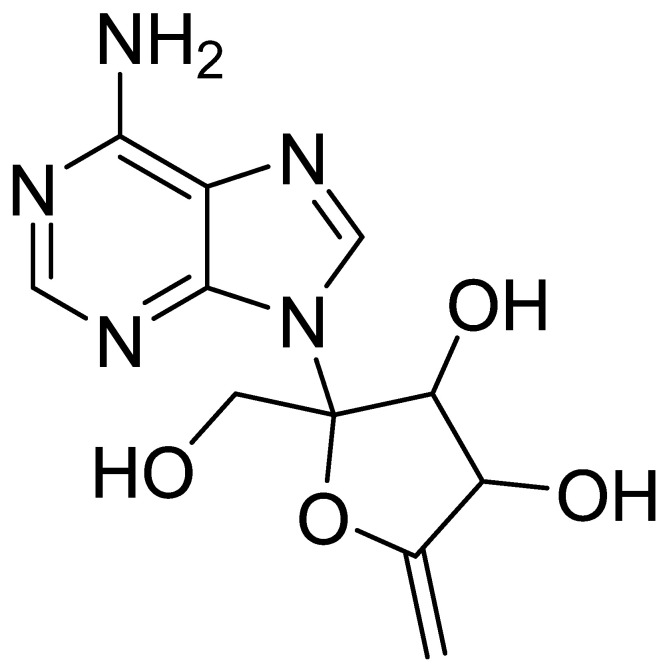
Structure of decoyinine.

Angustmycin A was initially extracted from *S. hygroscopicus* and an incorrect structure was allocated [38,39]. Consequently, the antibiotic decoyinine (DCY) was found to be similar with angustmycin A, and the right structure was determined to be 9-(6-deoxy-β-D-erythro-hex-5-enofuran-2-ulosyl) adenine (Figure 1) [40].

Previously, researchers investigated DCY’s effects as a nucleoside antibiotic, antimicrobial and antitumor [41,42]. DCY has been referred to as Lingfasu or Wugufengsu in China and its applications in botany and agronomy have been studied extensively [43]. DCY also promotes the rooting rate, root number and rooting scope of tissue-cultured plantlets of *Fructus momordicae* and *Arabidopsis thaliana* by increasing the level of endogenous indole-3-acetic acid [44]. DCY has been shown to enhance rice and maize yields while also improving disease tolerance [45]. A 50 mg L^−1^ DCY solution was applied to the Nanning rice variety “Baixiang 139,” which improved a number of physiological indices such as germination potential, germination rate, root length, bud length, leaf age, seedling base width, plant height, root number, fresh weight and dry matter accumulation [46]. Though it can affect both auxin and cytokinin regulatory pathways at the same time, it is vulnerable to mutants of auxin and cytokinin targets, suggesting that there could be new approaches to encourage growth and disease resistance [45]. However, it has only been occasionally recorded that DCY treatment can induce plant insect resistance. The ultimate impacts of DCY on insect performance were determined by the interaction of a positive effect resulting in the form of increased plant growth and a negative effect in the form of induced plant resistance [47].

Plants normally respond to herbivorous insect feeding with a series of chemical resistance responses. MDA is molecular indicator that shows the injury level of plant cell membrane and higher content of MDA as one of the physiological and biochemical responses of plants against insects [48]. A variety of stresses (biotic and abiotic) contribute to the rapid synthesis of reactive oxygen species (ROS), which triggers the plant immune system [49]. Enzymes, such as SOD and CAT, scavenge ROS molecules under steady-state conditions, and they are particularly effective in maintaining ROS balance [50]. PPO synthesizes insect-resistant secondary metabolites (phenols and polyphenols), and its activity changes in response to insect attack, similar to that of antioxidant enzymes [48,51]. The nutritional contents of host plants affect the growth and development of insect herbivores, soluble sugar being one of the most important sources of nutrients for herbivores. 

In this study, we tested the hypothesis that DCY treatment could induce a defense response in rice against SBPH. We studied the life cycle of SBPH on DCY treated rice and further investigated whether DCY treatment induces activity changes in rice defense related enzymes (CAT, SOD, POD, PAL and PPO) and MDA, H_2_O_2_ and soluble sugars in response to SBPH infestation duration. Such information may provide a better insight into DCY-mediated resistance to SBPH, which could improve rice insect pest management.

## 2. Materials and Methods

### 2.1. Plant and Insect

In the laboratory, SBPH was reared using the rice variety Wuyujing-3, provided by China National Rice Research Institute, Hangzhou, China. SBPH were reared continuously in the laboratory and were raised in plastic frame cages (50 cm (L): 50 cm (W): 30 cm (H)) in an artificial climate chamber (27 ± 1 °C, 70–80% (RH) and 14:10 h light: dark (L:D)) on paddy seedlings (10 ± 5 days) [52]. Cages were made of nylon mesh cloth, having a zippered entrance on one side. Rice seedlings were changed every 10–14 days to assure sufficient nutrition for SBPH [53].

### 2.2. DCY Treatments

Seeds were soaked for 24 h at 28 °C in various concentrations of DCY water solution (0, 25, 50 and 100 mg DCY L^−1^), then sprouted for another 24 h in a dark environment. The rice seedlings were individually placed inside glass tubes (diameter 2 cm, height 20 cm) after sprouting. All glass tubes were filled with nutritional soil (Jiangsu Xingnong Substrate Technology Co. Ltd., China). The rice seedlings were maintained in a growth chamber (27 ± 1 °C, 70–80% RH, and 14:10 h (L: D)). All rice seedlings were maintained free of insects until they were required. 

### 2.3. Life Table Parameters of SBPH

The freshly hatched SBPH nymphs were captured from a stock population with the help of an aspirator and placed individually into glass tubes (diameter 2 cm, height 20 cm) and raised on different DCY treated rice seedlings (10 ± 2 days after germination). Rice seedlings were changed every week until adult emergence. SBPH nymphs’ molting and mortality were observed daily. For each treatment, 50 replicates (one SBPH per seedling) were used. After adult emergence, one male and female of similar treatment were mated into a fresh glass tube containing the respective DCY treated paddy seedling. Every two days, the adult females were captured one by one with the help of an aspirator and transferred to new test tubes with corresponding DCY treated rice seedlings. The number of eggs were counted using a microscope and a needle to dissect rice seedlings until adult female death. Life table parameters were calculated with the following formulas: net reproductive rate: R_0_ = ∑ (l_x_m_x_), the mean generation time: T = ∑ (xl_x_m_x_)/∑ (l_x_m_x_), the intrinsic rate of increase: *r*_m_ = (lnR_0_)/T, finite rate of increase: *λ* = exp*^r^*^m^, the doubling time: DT = ln2/*r*_m_, x represents age in days, l_x_ represents the age-specific survival rate, m_x_ represents the age specific fecundity, and l_x_m_x_ represents age-specific maternity [54]. 

### 2.4. Determination of Biochemical and Physiological Indices

#### 2.4.1. Sample Collection and Preparation

To determine the amount of H_2_O_2_, MDA, soluble sugars and plant protective enzymes (CAT, SOD, POD, PAL and PPO) in 30-day old (control and DCY treated) rice plants were measured in response to SBPH infestation. A hundred rice plants for each treatment (0, 25, 50 and 100 mg DCY L^−1^) were grown in plastic buckets enclosed in cages under natural conditions, kept free of insects for one month. The plants were uprooted and their roots were washed with flowing tap water. Plants were then transferred into glass tubes (diameter 3 cm, length 28 cm) with nutritional solution (Kimura B solution) at the bottom in order to keep the plants alive. A sponge disc (diameter 3 cm, thickness 2 cm) was used to hold the rice plants in test tubes above the nutritional solution, then insects were transferred into the glass tubes and allowed to feed on DCY treated rice plants for different time periods. Another sponge disc was used to block the opening of the glass tubes to avoid insect escape. Test tubes having rice seedlings and insects were maintained in an artificial controlled environment (27 ± 1 °C, 70–80% (RH), and 14:10 h (L:D)), after specified insects feeding (0, 24, 48, 72, 96 (hpi)), the samples were collected from insects-exposed local (damaged) plant tissues and the remaining tissues were cut with scissors. The samples were immediately collected and carefully crushed in liquid nitrogen in a sterilized mortar using a pestle. The powder samples were moved to Eppendorf tubes with proper labeling and kept at −80 °C for subsequent analysis. For all treatments, the samples were collected 5 times. On each time period, sampling was repeated 5 times for different rice seedlings. Physiological and biochemical indices of rice were determined from already collected samples by preparing separate supernatant samples for each parameter assessment based on their individual diagnostic kits’ instructions.

#### 2.4.2. Measurement of MDA, H_2_O_2_ and Soluble Sugars

MDA is a biochemical marker which indicates the degree of membrane stress and injury [55]. The thiobarbituric acid method was used to determine MDA content [56] with an MDA testing kit (Nanjing Jiancheng Bioengineering Institute, Nanjing, China) and a Multiskan Spectrum (BioTek, Winooski, VT, USA). The ammonium molybdate spectrophotometric approach [57] was used to calculate H_2_O_2_ using an H_2_O_2_ analyzing tool (Nanjing Jiancheng Bioengineering Institute, Nanjing, China). Soluble sugars of rice seedlings were calculated using the anthrone colorimetry procedure [58] by exploiting the appropriate analyzing tool (Nanjing Jiancheng Bioengineering Institute, Nanjing, China). For all four treatments (0, 25, 50 and 100 mg DCY L^−1^), five seedlings were destructively harvested at five time intervals (i.e., 0, 24, 48, 72 and 96 (hpi)), giving a total of 100 seedlings samples.

#### 2.4.3. Enzymes Activities Tests 

In a recent study, the same enzyme activity testing techniques utilized by Han [51] were applied. Antioxidant enzymes (CAT, SOD and POD) and secondary metabolite synthases (PAL and PPO) activities of paddy plants were determined with respective analyzing kits (Nanjing Jiancheng Bioengineering Institute, Nanjing, China). The activity of CAT was determined using the ammonium molybdate spectrophotometric technique [58]. A spectrometer (UV-2000, UNICO, Shanghai, China) was used to assess the activity of POD following the change of absorption at 420 nm due to guaiacol oxidation [59]. The activity of SOD was measured using 2-(4-iodophenyl)-3-iodophenyl)-3-iodophenyl-3-iodophenyl)-3-iodophenyl)-3-iod (4-nitrophenyl)-5% (2,4-disulfophenyl) Tetrazolium-2H (WST-1) [60]. The activities of PPO and PAL were assessed using the techniques of Cai [61]. For all four treatments (0, 25, 50 and 100 mg DCY L^−1^), five seedlings were destructively harvested at five time intervals (i.e., 0, 24, 48, 72 and 96 (hpi)), giving a total of 100 seedlings samples.

### 2.5. Statistical Analysis

The survival rate of SBPH was analyzed via log-rank test. Biological parameters of SBPH were analyzed by the Tukey’s multiple-range test. TWOSEX-MSChart was carried out for analyzing the population life table parameters [62]. To obtain standard errors and variances of population parameters, bootstrap technique was utilized with 100,000 bootstrap replicates. Means and differences between DCY treatments were calculated at 5% significance level using paired bootstrap test. One-way ANOVA was used to assess differences in oviposition of SBPH on rice seedlings, as well as survival, followed by Tukey’s honestly significant difference (HSD) test. The data of enzyme activities and concentrations of MDA, H_2_O_2_ and soluble sugars were subjected to generalized linear model (GLM) for the effects of DCY treatment, SBPH infestation duration and the interactions between the two treatments. Means were separated by Tukey’s honestly significant difference (HSD) test (*p* < 0.05). SPSS 16.0 was used for all statistical analyses (SPSS Inc., Chicago, IL, USA). 

## 3. Results

### 3.1. Biological Life Table Parameters of SBPH

SBPH survivals were investigated in both the control and DCY treated rice treatments (Table 1), indicating that the number of eggs laid by SBPH fed on DCY treated rice plants was significantly lower at rice seedling stage. In comparison to control seedlings, the DCY treatment had no effect on nymphal duration (male and female), adult longevity (male and female), pre-oviposition period and oviposition period. The fecundity, on the other hand, was significantly lowered by 40.27% (Table 1). 

### 3.2. Population Life Table Parameters of SBPH

In contrast to the control, the intrinsic rate of natural increase (*r*_m_) and the finite capacity of increase (*λ*) in SBPH in the DCY50 and DCY100 treatments were significantly lowered, but the doubling time (DT) was significantly higher. The net reproductive rate (*R*_0_) of SBPH fed on DCY100 rice was significantly lowered by 43.63%. The mean generation time (*T*) was significantly changed in DCY50 and DCY100 (Table 2).

### 3.3. Effects of DCY Treatment on Rice MDA and H_2_O_2_ Concentration against SBPH 

**Figure 2:**
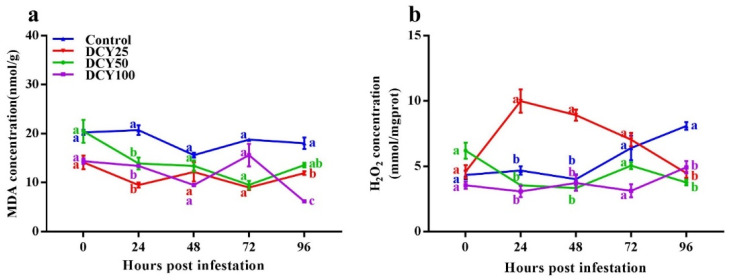
Concentrations of chemical indices in rice leaf sheaths in response to DCY treatment and SBPH infestation. (**a**) MDA, (**b**) H_2_O_2_. Data represent means ± standard error of three replicates. Significant difference between DCY and control plants is indicated by letters (a,b,c) (*p* < 0.05, Tukey’s HSD test).

Activities of MDA and H_2_O_2_ were studied in response of DCY treatment and SBPH infestation duration (Table 3 and Figure 2). GLM showed that DCY treatment, SBPH infestation time and their interaction all significantly influenced MDA concentration (Table 3). MDA concentrations in both DCY treated and control rice plants responded negatively to SBPH infestation. Without SBPH infestation, the differences in MDA concentrations were not significantly different between DCY treated and control plants at 0 hpi (Figure 2a; *p* < 0.05). In subsequent time periods (24, 48, 72 and 96 hpi) with SBPH infestation, MDA concentrations were decreased and showed a gradual decreasing temporal pattern (*df* = 4, χ^2^ = 190.660, *p* < 0.001). Among the treatments, MDA concentrations were significantly lower in DCY 25 (9.51 ± 0.48 nmol/g) and DCY 100 (6.17 ± 0.12 nmol/g) in comparison with control at 24 and 96 hpi, respectively (*df* = 3, χ^2^ = 504.617, *p* < 0.001). 

GLM regarding H_2_O_2_ showed that DCY treatment and their interaction significantly affected H_2_O_2_ concentration (Table 3). H_2_O_2_ concentrations in both DCY treated and control plants responded differently to SBPH infestation. Without SBPH infestation, the differences in H_2_O_2_ concentrations were not significant between DCY treated and control plants at 0 hpi (Figure 2b; *p* < 0.05). In subsequent time periods (24, 48, 72 and 96 hpi) with SBPH infestation, H_2_O_2_ concentrations sharply increased in DCY 25 at 24 hpi, while a gradual decreasing temporal pattern was observed in DCY 50 and DCY 100 (*df* = 4, χ^2^ = 4.540, *p* = 0.338). Among the treatments, H_2_O_2_ concentrations were significantly lower in DCY 100 (3.08 ± 0.43 mmol/mgprot.), DCY 50 (3.34 ± 0.22 mmol/mgprot.) and DCY 50 (3.76 ± 0.21 mmol/mgprot.) in comparison with control at 24, 48 and 96 hpi, respectively (*df* = 3, χ^2^ = 94.482, *p* < 0.001). 

### 3.4. Effects of DCY Treatment on Rice Antioxidant Enzymes against SBPH

**Figure 3:**
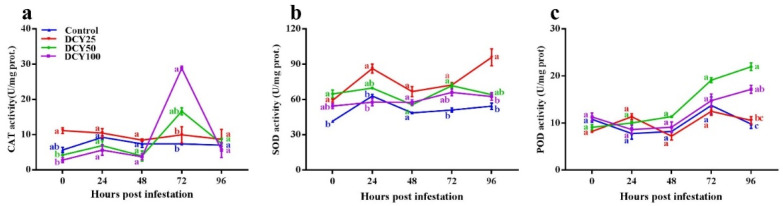
Activities of antioxidant enzymes in rice leaf sheaths in response to DCY treatment and SBPH infestation. (**a**) CAT, (**b**) SOD, (**c**) POD. Data represent means ± standard error of three replicates. Significant difference between DCY and control plants is indicated by letters (a,b,c) (*p* < 0.05, Tukey’s HSD test).

GLM showed that DCY treatment, SBPH infestation time and their interaction all significantly influenced CAT concentrations (Table 3). CAT concentrations in both DCY treated and control rice plants responded positively to SBPH infestation. Without SBPH infestation, the CAT concentration was significantly different between DCY treated and control plants at 0 hpi (Figure 3a; *p* < 0.05). In succeeding time periods (24, 48, 72 and 96 hpi) with SBPH infestation, CAT concentrations were steadily increased from 0 to 48 hpi, thereafter abruptly declining (*df* = 4, χ^2^ = 669.002, *p* < 0.001). Among the treatments, CAT concentrations were significantly higher in DCY 100 (28.82 ± 0.59 U/mg prot.) in comparison with control at 72 hpi (*df* = 3, χ^2^ = 57.399, *p* < 0.001). 

GLM regarding SOD showed that DCY treatment, SBPH infestation time and their interaction all significantly affected SOD concentration (Table 3). SOD concentrations in both DCY treated and control rice plants responded positively to SBPH infestation. Without SBPH infestation, the SOD concentration was significantly different between DCY treated and control plants at 0 hpi (Figure 3b; *p* < 0.05). In succeeding time periods (24, 48, 72 and 96 hpi) with SBPH infestation, SOD concentrations steadily increased and showed a regular temporal pattern (*df* = 4, χ^2^ = 2169.256, *p* < 0.001). Among the treatments, SOD concentrations were significantly higher in DCY 25 (86.30 ± 3.83 U/mg prot.), DCY 25 (72.31 ± 2.19 U/mg prot.) and DCY 25 (95.87 ± 7.19 U/mg prot.) in comparison with control at 24, 72 and 96 hpi, respectively (*df* = 3, χ^2^ = 4713.175, *p* < 0.001). 

GLM showed that DCY treatment, SBPH infestation time and their interaction all significantly influenced POD concentration (Table 3). POD concentrations in both DCY treated and control rice plants responded positively to SBPH infestation. Without SBPH infestation, the POD concentration was not significantly different between DCY treated and control plants at 0 hpi (Figure 3c; *p* < 0.05). In succeeding time periods (24, 48, 72 and 96 hpi) with SBPH infestation, POD concentrations increased (*df* = 4, χ^2^ = 445.979, *p* < 0.001). Among the treatments, POD concentrations were significantly higher in DCY 50 (21.97 ± 0.81 U/mg prot.) in comparison with control at 96 hpi (*df* = 3, χ^2^ = 191.062, *p* < 0.001). 

### 3.5. Effects of DCY Treatment on Rice Synthases Enzymes against SBPH

**Figure 4:**
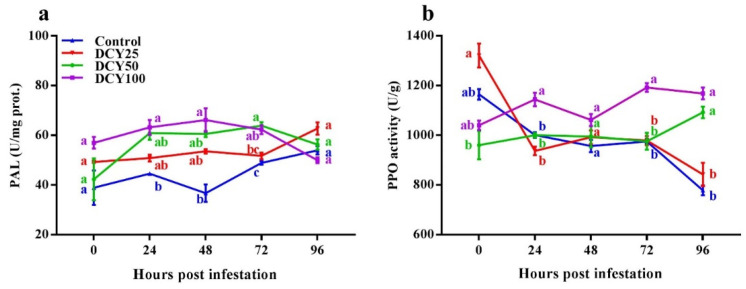
Activities of synthases enzymes of secondary metabolites in rice leaf sheaths in response to DCY treatment and SBPH infestation. (**a**) PAL, (**b**) PPO. Data represent means ± standard error of three replicates. Significant difference between DCY and control plants is indicated by letters (a,b,c) (*p* < 0.05, Tukey’s HSD test).

PAL and PPO activities were investigated in response to DCY treatment and SBPH infestation duration (Table 3 and Figure 4). GLM showed that DCY treatment, SBPH infestation time and their interaction all significantly influenced PAL concentrations (Table 3). PAL concentrations in both DCY treated and control plants responded positively to SBPH infestation. Without SBPH infestation, the PAL concentration was not significantly different between DCY treated and control plants at 0 hpi (Figure 4a; *p* < 0.05). In the following time periods (24, 48, 72 and 96 hpi) with SBPH infestation, PAL concentrations increased (*df* = 4, χ^2^ = 739.560, *p* < 0.001). Among the treatments, PAL concentrations were significantly higher in DCY 100 (63.24 ± 3.00 U/mg prot.), DCY 100 (66.17 ± 4.72 U/mg prot.) and DCY 50 (63.89 ± 1.43 U/mg prot.) in comparison with control at 24, 48 and 72 hpi, respectively (*df* = 3, χ^2^ = 1928.716, *p* < 0.001). 

GLM regarding PPO showed that DCY treatment, SBPH infestation time and their interaction all significantly influenced PPO concentrations (Table 3). PPO concentrations in both DCY treated and control plants responded positively to SBPH infestation. Without SBPH infestation, the PPO concentration was significantly different between DCY treated and control plants at 0 hpi (Figure 4b; *p* < 0.05). In following time periods (24, 48, 72 and 96 hpi) with SBPH infestation, PPO concentrations increased (*df* = 4, χ^2^ = 154,459.437, *p* < 0.001). Among the treatments, PPO concentrations were significantly higher in DCY 100 (1144.22 ± 27.45 U/g), DCY 100 (1192.44 ± 17.44 U/g) and DCY 100 (1168.44 ± 23.62 U/g) in comparison with control at 24, 72 and 96 hpi, respectively (*df* = 3, χ^2^ = 183,694.459, *p* < 0.001). 

### 3.6. Effects of DCY Treatment on Rice Soluble Sugar against SBPH

**Figure 5:**
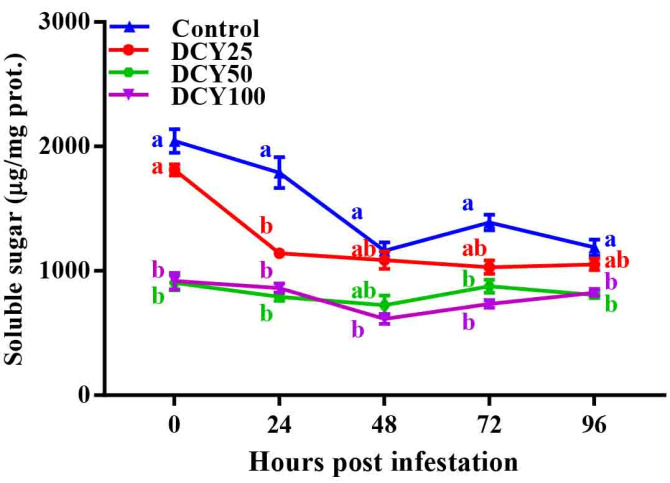
Concentrations of soluble sugar content in rice leaf sheaths in response to DCY treatment and SBPH infestation. Data represent means ± standard error of three replicates. Significant difference between DCY and Control plants is indicated by letters (a,b) (*p* < 0.05, Tukey’s HSD test).

GLM regarding soluble sugars showed that DCY treatment, SBPH infestation time and their interaction all significantly influenced sugar concentrations (Table 3). Soluble sugar contents in both DCY treated and control plants responded negatively to SBPH infestation. Without SBPH infestation, the soluble sugar contents were significantly different between DCY treated and control plants at 0 hpi (Figure 5; *p* < 0.05). In subsequent time periods (24, 48, 72 and 96 hpi) with SBPH infestation, soluble sugar contents decreased (*df* = 4, χ^2^ = 2,050,377.501, *p* < 0.001). Among the treatments, soluble sugar contents were significantly lower in DCY 50 (791.65 ± 32.86 µg/mg prot.), DCY 100 (614.44 ± 40.17 µg/mg prot.), DCY 100 (734.20 ± 29.85 µg/mg prot.) and DCY 50 (805.41 ± 23.89 µg/mg prot.) in comparison with control at 24, 48, 72 and 96 hpi, respectively (*df* = 3, χ^2^ = 5,400,485.652, *p* < 0.001). 

## 4. Discussions 

Colonization of host plant roots with beneficial bacteria can induce ISR [63]. ISR is facilitated via beneficial soil microbes which usually rely on priming [64]. Priming with microbes is an excellent approach to conserve energy by suppressing basal plant resistance [65,66,67,68]. Priming can increase resistance against herbivore and pathogen incidence in rice and other crops [69]. Primed plants demonstrate a rapid and greater cellular defense initiation against insect and pathogen attack in order to enhance plant defense level [70]. Induced resistance might be utilized as a useful technique in pest management to reduce pesticide applications against insect pests. In particular, induced host plant resistance to insects can be deployed by using chemical elicitors of secondary metabolites that impart insect resistance. Induced response elicitors can be applied to agricultural plants to strengthen the natural defense mechanism against herbivore injury and can serve as a component of integrated pest management for long-term agricultural production [71].

The present study was initiated with the same concept to evaluate the effects of DCY (secondary metabolites elicitor) on rice against SBPH. *Bacillus velezensis* YC7010 is an endophytic bacterium that promotes induced defenses against BPH, when the roots of rice seedlings are treated [16]. Prior research revealed that a synthetic chemical elicitor (gibberellins, 4-fluorophenoxyacetic acid) boosted rice resistance to piercing–sucking insect pests [72,73]. To better understand the impacts of DCY on SBPH, we measured the oviposition performance of SBPH on rice seedlings in the lab and discovered that DCY reduced SBPH fecundity (Table 1). A previous study also revealed that the exogenous application of abscisic acid promoted rice resistance against BPH by reducing its fecundity [74]. *Azotobacter chroococcum* boosts maize yields while decreasing *Mythimna separata* pupation rate and fecundity [75]. The chemical elicitor, gibberellin, had a negative impact on the survival, development and reproduction of BPH female adults [72]. Although DCY had no effect on SBPH survival, the fecundity of SBPH was significantly reduced in laboratory (Table 1).

The population life table parameters of SBPH, such as *r*_m_, *R*_0_, DT and λ, were greatly influenced by rice with DCY treatment (Table 2). Adult emergence, adult longevity and the fecundity of *Spodoptera litura* were significantly reduced by *Streptomyces hydrogenans* DH16 [76]. SBPH showed reduced female ratio and fecundity when treated with bacterial pesticide, *Beauveria bassiana sensu lato* isolate NJBb2101, which in turn retarded the growth and development of SBPH along with enhanced pesticide susceptibility [77]. So, we assumed that DCY could enhance rice resistance to SBPH. Insect behaviors and life table parameters were regulated by environmental factors such as humidity, temperature and morphological and chemical components of host plants, including sugars, nitrogen, enzymes and secondary metabolites [78,79].

According to our findings, DCY tends to interfere with a variety of defense-related plant defenses that use signaling channels to regulate a variety of physiological and biochemical activities. Excess MDA can damage cell membranes. Conferring to our results, MDA levels in DCY treated plants decreased from 24 to 96 hpi (Figure 2a). H_2_O_2_ triggers a series of events in plants that result in the activation of defense genes, defending them against herbivores by activating chloroplast and peroxisome autophagy. H_2_O_2_ levels in DCY treated rice plants were found to be substantially lower, according to our findings (Figure 2b). H_2_O_2_ has been shown to catalyze the accumulation of phenolic polymers in rice parenchyma cells and has been linked to reduced feeding ability in *Sogatella furcifera* female adults as well as lower survival rates in other piercing and sucking herbivores [72].

SOD and CAT can also develop a mechanical barrier to strengthening plant cell wall structures to enhance herbivore resistance [80,81]. In this study, DCY boosted the activities of CAT and SOD (Figure 3a,b), which is parallel with the findings of Harun-Or-Rashid [16]; he observed that at 48 hpi against BPH, the activities of POD, PAL and PPO were considerably greater in bacteria YC7010 treated rice than in untreated control plants. In our study, the activity of POD was enhanced against SBPH infestation in DCY treated plants with time, and higher significant activity was observed at 96 hpi (Figure 3c). The same trend of enhanced activities for PAL and PPO was observed in DCY treated rice plants against SBPH (Figure 4a,b). PAL induced resistance against BPH in rice by regulating biosynthesis and accumulation of salicylic acid and lignin via Phenylalanine ammonia-lyase pathway [82]. In our study, PAL activity was stimulated subsequently on SBPH infestation, and it enhanced significantly in DCY treated plants at 24 hpi (Figure 4a). PPO catalyzes the oxidation of phenols or polyphenols to synthesize quinones, which can reduce herbivorous insect nutrition absorption. PPO activity was elicited shortly after SBPH infestation, and it increased considerably in DCY treated plants (Figure 4b).

Rice pest resistance is mostly influenced by soluble sugar. In a current study, SBPH infestations have been demonstrated to impact sugar metabolism in rice plants [83]. The addition of DCY caused a considerable decline in soluble sugar content, according to our findings (Figure 5). Similar results of low sugar content were detected in the resistant rice variety against higher quantities of total phenols [84]. In a recent study, the phenolic enzymatic activities were enhanced by DCY treatment. In contrast, Jinggangmycin, a fungicide spray, was used to control rice sheath blight (*Rhizoctonia solani*), which in turn increased rice plant soluble sugars (sucrose and glucose), sugar metabolism gene expression and resulting BPH population expansion [85].

In short, the results obtained regarding physiological and biochemical indices of rice plant via GLM showed that all the indices were significantly affected with DCY treatment, while the results regarding SBPH infestation time showed that all the physiological and biochemical indices of rice plant were significantly affected with time, except H_2_O_2_ (*df* = 4, χ^2^ = 4.540_,_ *p* = 0.338) (Table 3). A similar approach of GLM analysis was followed to study the mortality of *Rhyzopertha dominica*, *Sitophilus oryzae*, *Oryzaephilus surinmaneis* and *Tribolium castaneum* against different phosphine concentrations and insect exposure time intervals [86]. DCY application could be the possible reason in the priming of ROS, antioxidant enzymes, secondary metabolic enzymes and sugar metabolism mechanisms, which might explain how rice resistance against SBPH is augmented. 

## 5. Conclusions

In conclusion, our results showed that DCY treatment has significantly lowered the fecundity of SBPH by 40.27%. In addition, it has the ability to significantly alter the activities of antioxidant enzymes (CAT, SOD and POD) and synthases for secondary metabolites (PAL and PPO), MDA, H_2_O_2_ and soluble sugars against SBPH infestation. Our findings demonstrate that DCY has the potential to become a novel bio-pesticide for SBPH management. In future research, the impacts of DCY on defense-related gene expression in rice should be thoroughly considered. 

## Figures and Tables

**Table 1:** Biological parameters of SBPH fed on rice under laboratory conditions.

Parameters	Control	DCY25	DCY50	DCY100
First instar (days)	1.94 ± 0.023 a	1.84 ± 0.022 a	2.06 ± 0.022 a	2.06 ± 0.019 a
Second instar (days)	2.26 ± 0.022 a	2.25 ± 0.017 a	2.33 ± 0.019 a	2.60 ± 0.021 a
Third instar (days)	2.95 ± 0.024 a	2.77 ± 0.022 a	2.77 ± 0.025 a	2.60 ± 0.021 a
Fourth instar (days)	2.62 ± 0.025 a	2.59 ± 0.024 a	2.54 ± 0.020 a	2.40 ± 0.023 a
Fifth instar (days)	3.03 ± 0.037 a	2.71 ± 0.023 a	2.72 ± 0.039 a	2.59 ± 0.029 a
Total nymphal duration of males (days)	12.67 ± 0.120 a	11.31 ± 0.109 a	11.70 ± 0.073 a	11.80 ± 0.107 a
Total nymphal duration of females (days)	12.00 ± 0.146 a	11.96 ± 0.075 a	13.00 ± 0.109 a	12.17 ± 0.067 a
Male longevity (days)	20.78 ± 0.215 a	19.83 ± 0.231 a	19.34 ± 0.204 a	21.63 ± 0.302 a
Female longevity (days)	24.08 ± 0.256 a	24.92 ± 0.260 a	25.60 ± 0.265 a	25.53 ± 0.365 a
Pre-oviposition period (days)	5.80 ± 0.038 a	5.90 ± 0.039 a	5.69 ± 0.041 a	6.48 ± 0.032 a
Oviposition period (days)	18.05 ± 0.211 a	18.27 ± 0.211 a	18.73 ± 0.196 a	18.20 ± 0.248 a
Fecundity (eggs per female)	144.23 ± 2.312 a	97.00 ± 1.323 b	98.44 ± 1.608 b	86.14 ± 1.386 b

a,b Means ± standard error in the same rows followed by the same letters are not significantly different among different rice treatments at *p* < 0.05 by using Tukey’s multiple-range test.

**Table 2:** Population life table parameters of SBPH fed on rice under laboratory conditions.

Parameters	Control	DCY25	DCY50	DCY100
*r*_m_	0.13 ± 0.006 a	0.11 ± 0.005 ab	0.10 ± 0.006 b	0.09 ± 0.006 b
*R*_0_	49.04 ± 7.846 a	37.31 ± 6.207 a	30.380 ± 5.739 a	27.64 ± 5.067 b
*T* (days)	30.74 ± 0.708 b	31.48 ± 0.498 b	33.56 ± 0.722 a	34.09 ± 0.703 a
DT (days)	5.47 ± 0.270 c	6.03 ± 0.289 bc	6.81 ± 0.428 ab	7.12 ± 0.435 a
*λ*	1.13 ± 0.007 a	1.12 ± 0.006 ab	1.11 ± 0.007 bc	1.10 ± 0.006 c

*r*_m_: the intrinsic rate of natural increase, *R*_0_: the net reproductive rate, *T*: the mean generation time, DT: the doubling time, *λ*: the finite capacity of increase. Means ± standard errors were estimated using the bootstrap technique with 100,000 re-samplings. Differences between two treatments were compared using a paired bootstrap test implemented in TWOSEX-MSChart. The means in the same rows followed by different lowercase letters (a,b,c) indicate significant differences between treatments (*p* < 0.05).

**Table 3:** Generalized linear model for significance effect (*p* value) of DCY treated plants, SBPH infestation duration on plant protective enzymes activities and concentrations of MDA, H_2_O_2_ and soluble sugar.

Physiological Indices		*df*	χ^2^	*p*
MDA	DCY treatment (A)	3	504.617	<0.001
	Infestation time (B)	4	190.660	<0.001
	Interaction (A × B)	12	280.782	<0.001
H_2_O_2_	DCY treatment (A)	3	94.482	<0.001
	Infestation time (B)	4	4.540	0.338
	Interaction (A × B)	12	129.577	<0.001
CAT	DCY treatment (A)	3	57.399	<0.001
	Infestation time (B)	4	669.002	<0.001
	Interaction (A × B)	12	798.850	<0.001
SOD	DCY treatment (A)	3	4713.175	<0.001
	Infestation time (B)	4	2169.256	<0.001
	Interaction (A × B)	12	1942.936	<0.001
POD	DCY treatment (A)	3	191.062	<0.001
	Infestation time (B)	4	445.979	<0.001
	Interaction (A × B)	12	252.641	<0.001
PAL	DCY treatment (A)	3	1928.716	<0.001
	Infestation time (B)	4	739.560	<0.001
	Interaction (A × B)	12	1568.780	<0.001
PPO	DCY treatment (A)	3	183,694.459	<0.001
	Infestation time (B)	4	154,459.437	<0.001
	Interaction (A × B)	12	553,054.282	<0.001
Soluble sugar	DCY treatment (A)	3	5,400,485.652	<0.001
	Infestation time (B)	4	2,050,377.501	<0.001
	Interaction (A × B)	12	1,305,630.400	<0.001

DCY treatment (0, 25, 50 and 100 mg DCY L^− 1^) water solution, SBPH infestation duration: 0, 24, 48, 72 and 96 h. Significant differences at *p* < 0.05 by using Chi-square test.

## Data Availability

Data are contained within the article.
